# SPECTRUM: Early clinical experience from the first global real-world study of aflibercept 8 mg in patients with diabetic macular oedema

**DOI:** 10.1038/s41433-026-04659-y

**Published:** 2026-07-11

**Authors:** Clare Bailey, Marion R. Munk, Varun Chaudhary, Clemens Lange, Hassiba Oubraham, Peter Morgan-Warren, Helmut Allmeier, Tobias Machewitz, Sarah Schlief, Rose Gilbert, Susanne Oesch, Martin Kirchner, Zoran Hasanbasic, Paolo Lanzetta

**Affiliations:** 1 Department of Ophthalmology, University Hospitals Bristol and Weston NHS Foundation Trust, Bristol, UK; 2 Augenarzt Praxisgemeinschaft Gutblick AG, Pfäffikon, Switzerland; 3 Department of Ophthalmology, University Hospital Bern, Bern, Switzerland; 4 Northwestern University, Feinberg School of Medicine, Chicago, IL USA; 5 Department of Surgery, McMaster University, Hamilton, ON Canada; 6 Eye Center, Faculty of Medicine, Albert-Ludwig University Freiburg, Freiburg, Germany; 7 Department of Ophthalmology, St Franziskus Hospital, Münster, Germany; 8 Centre OPHTA-45, Montargis, France; 9 Bayer Consumer Care AG, Basel, Switzerland; 10 Bayer AG, Berlin, Germany; 11 Bayer AG, Leverkusen, Germany; 12 Department of Medicine–Ophthalmology, University of Udine, Udine, Italy; 13 Istituto Europeo di Microchirurgia Oculare (IEMO), Udine, Italy; 14 Retina and Macula Specialists Miranda, Sydney, NSW Australia; 15 Specialist Eye Group, Melbourne, Australia; 16 Sydney Retina Clinic and Day Surgery, Sydney, NSW Australia; 17 Vision Eye Institute Boronia, Melbourne, VIC Australia; 18 South West Retina, Liverpool, NSW Australia; 19 Marsden Eye Institute Parramatta, Parramatta, NSW Australia; 20 Sydney West Retina, Sydney, NSW Australia; 21 Retina Specialists Victoria Warragul, Warragul, VIC Australia; 22 Samad Ophthalmology, Halifax, NS Canada; 23 UHN, Toronto, ON Canada; 24 Retine de l’est, Montreal, QC Canada; 25 Eye Health MD, Montreal, QC Canada; 26 Trimed Eye Centre, Orillia, ON Canada; 27 Toronto Retina, Toronto, Canada; 28 VRMTO, Toronto, ON Canada; 29 Mississauga Retina Institute, Mississauga, ON Canada; 30 Retina Centre of Ottawa, Ottawa, ON Canada; 31 Retina Surgical Associates, New Westminster, BC Canada; 32 Uptown Eye Specialists, Vaughan, ON Canada; 33 SJHC London Eye, London, ON Canada; 34 RSJH, Hamilton, Canada; 35 Vancouver General Hospital, Vancouver, BC Canada; 36 Aalborg University Hospital - Eye Department, Aalborg, Denmark; 37 Rigshospitalet Glostrup - Afdeling for Øjensygdomme, Glostrup, Denmark; 38 Hospital of Southern Jutland - Department of Eye Diseases, Esbjerg, Denmark; 39 Tampere University Hospital, Tampere, Finland; 40 Helsinki Retina Research Group, Helsinki, Finland; 41 Institut d’ophtalmologie Sourdille Atlantique, Saint-Herblain, France; 42 Hôpital Lariboisière Fernand-Widal, Paris, France; 43 Hôpital Avicenne, Bobigny, France; 44 Centre Ophtalmologique Maison rouge, Strasbourg, France; 45 Hôpital Edouard Herriot HCL, Lyon, France; 46 Paris Retina Vision, Paris, France; 47 CHIC Créteil, Créteil, France; 48 Pole Vision Val d’Ouest, Lyon, France; 49 Institut Ophtalmologique de l’Ouest, Nantes, France; 50 Centre Monticelli Paradis, Marseille, France; 51 Ophta 45, Loiret, France; 52 Centre Ophtalmologique Saint Exupéry, Lyon, France; 53 Centre Ophtalmologie des Arceaux, Montpellier, France; 54 Clinique Mathilde, Rouen, France; 55 Centre d’Exploration de la rétine Kléber, Lyon, France; 56 Hôpital de la Croix Rousse, Lyon, France; 57 Centre Rétine Gallien, Bordeaux, France; 58 CHU de Bordeaux, Bordeaux, France; 59 Centre Opthalmologie Sorbonne Saint Michel, Paris, France; 60 Centre d’Ophtalmologie du Dauphiné, Grenoble, France; 61 Centre Aix Vision, Aix en Provence, France; 62 Centre d’Exploration de l’Odeon, Paris, France; 63 Clinique des yeux, Bordeaux, France; 64 CHU François Mitterrand Dijon, Dijon, France; 65 Centre Ophtalmologique d’Imagerie et de Laser, Paris, France; 66 CHU Pasteur, Nice, France; 67 CHU amiens, Amiens, France; 68 Clinique Honoré Cave, Montauban, France; 69 Clinique de l’Union, Saint Jean, France; 70 Augenblick Rheinland GmbH, Brühl, Germany; 71 Augenklinik Uniklinikum Würzburg, Würzburg, Germany; 72 1Universitätsmedizin Greifswald, Klinik und Poliklinik für Augenheilkunde, Greifswald, Germany; 73 Augenzentrum Berliner Ring, Würzburg, Germany; 74 Augenklinik am Wittenbergplatz, Berlin, Germany; 75 Diakonie Klinikum Dietrich Bonhoeffer, Neubrandenburg, Germany; 76 Augenklinik Universitätsklinikum Heidelberg, Heidelberg, Germany; 77 Augenzentrum am St. Franziskus-Hospital, Münster, Germany; 78 Costin Mihaescu, Würzburg, Germany; 79 Visualeins GmbH MVZ für Augenheilkunde, Osnabrück, Germany; 80 Marienhospital Osnabrück, Osnabrück, Germany; 81 St. Elisabeth-Krankenhaus GmbH, Katharinenberg, Germany; 82 Rund ums Auge GbR, Grefrath, Germany; 83 Sankt-Gertrauden-Krankenhaus GmbH Abteilung Augenheilkunde, Berlin, Germany; 84 Augenklinik Petrisberg (Medical eye research xperts Institut GmbH), Trier, Germany; 85 Augenklinik und Poliklinik, Universitätsmedizin Mainz, Mainz, Germany; 86 ASKLEPIOS Klinik Nord, Hamburg, Germany; 87 Universitätsklinikum Regensburg, Regensburg, Germany; 88 Smile Eyes Augenärzte, MVZ Augenärzte am Airport, Frankfurt am Main, Germany; 89 Charité Berlin, Campus Benjamin Franklin, Klinik für Augenheilkunde, Berlin, Germany; 90 Makula Center Augsburg, Südblick Augenzentren, Augsburg, Germany; 91 Augenzentrum im Medizeum, Mainz, Germany; 92 BeyondEye GmbH, Bonn, Germany; 93 Universitätsklinikum Aachen, Augenheilkunde, Aachen, Germany; 94 Kadina Research Zschopau, Zschopau, Germany; 95 Stiftung Pius - Hospital Universitätsklinik für Augenheilkunde, Oldenburg, Germany; 96 Augenzentrum Frankfurt, Frankfurt am Main, Germany; 97 Universitätsklinikum Köln, Augenheilkunde, Cologne, Germany; 98 MVZ Augenheilkunde Mitteldeutschland GmbH, Leipzig, Germany; 99 Department für Augenheilkunde, Universitätsklinikum Tübingen, Tübingen, Germany; 100 Gemeinschaftspraxis für Augenheilkunde, Munich, Germany; 101 MVZ der Klinik Dardenne GmbH Makulazentrum, Bonn, Germany; 102 Klinikum Chemnitz, Augenheilkunde, Chemnitz, Germany; 103 Philipps-Universität Marburg, Klinik für Augenheilkunde, Margurg, Germany; 104 Ospedale Generale Regionale “F. Miulli”, Acquaviva delle Fonti, Italy; 105 Ospedale Maggiore di Trieste, Trieste, Italy; 106 Ospedale Molinette, Turin, Italy; 107 Policlinico Tor Vergata, Rome, Italy; 108 Ospedale Monaldi, Naples, Italy; 109 S. Raffaele, Milan, Italy; 110 Ospedale Maggiore Policlinico di Milano, Milan, Italy; 111 Ospedale Sacco di Milano, Milan, Italy; 112 Ospedale SS Annunziata, Chieti, Italy; 113 Ospedale Parini, Aosta, Italy; 114 Ospedale Umberto I di Ancona, Ancona, Italy; 115 Policlinico “Paolo Giaccone”, Palermo, Italy; 116 Presidio Gaspare Rodolico, Catania, Italy; 117 Ospedale Universitario Santa Maria della Misericordia, Udine, Italy; 118 Ospedale Policlinico San Martino, Genoa, Italy; 119 Aoyagi Eye Clinic, Tokyo, Japan; 120 Osaka University Hospital, Osaka, Japan; 121 Fujimura Arimatsu Eye Clinic, Nagoya, Japan; 122 Fujiwara Eye Clinic, Kyoto, Japan; 123 Muramatsu Eye Clinic, Hamamatsu, Japan; 124 Otsuka Eye Clinic, Tokyo, Japan; 125 Chukyo Eye Clinic, Nagoya, Japan; 126 Chukyo Hospital, Nagoya, Japan; 127 Kaneda Eye Clinic, Osaka, Japan; 128 Yokkaichi Municipal Hospital, Yokkaichi, Japan; 129 Nagata Eye Clinic, Fukuoka, Japan; 130 Kimura Eye Hospital, Hiroshima, Japan; 131 Kozawa Eye Hospital and Diabetes Center, Gifu, Japan; 132 Miyake Eye Hospital, Nagoya, Japan; 133 Sannoudai Hospital Ganka Naika Clinic, Yokohama, Japan; 134 Yamagata University Hospital, Yamagata, Japan; 135 Fuchu Eye Center, Fuchu, Japan; 136 Hirota Eye Clinic, Osaka, Japan; 137 Kitano Hospital, Osaka, Japan; 138 Iida Municipal Hospital, Iida, Japan; 139 Kimitsu Chuo Hospital, Kimitsu, Japan; 140 Mikawa Eye Clinic, Okazaki, Japan; 141 Nagoya Medical Center, Nagoya, Japan; 142 Sato Ganka Clinic, Sendai, Japan; 143 Kobe University Hospital, Kobe, Japan; 144 Sugita Eye Hospital, Kumamoto, Japan; 145 Takagi Ophthalmic Hospital, Nagoya, Japan; 146 Takasu Eye Clinic, Toyohashi, Japan; 147 Nakatake Eye Clinic, Fukuoka, Japan; 148 Yamane Eye Clinic, Matsue, Japan; 149 Omiya Nanasato Eye Institute, Saitama, Japan; 150 Sapporo Kato Ophthalmology Clinic, Sapporo, Japan; 151 UMCG, Groningen, The Netherlands; 152 Erasmus MC, Rotterdam, The Netherlands; 153 ETZ, Tilburg, The Netherlands; 154 Jeroen Bosch Ziekenhuis, ‘s-Hertogenbosh, The Netherlands; 155 Maastricht UMC, Maastricht, The Netherlands; 156 Radboud UMC, Nijmegen, Netherlands; 157 LUMC, Leiden, The Netherlands; 158 Drammen Sykehus, Øyeavdelingen, Vestre Viken HF, Drammen, The Netherlands; 159 Sykehuset Østfold, Moss - Eye Department, Moss, The Netherlands; 160 Unidade Local de Saúde da Região de Leiria, Leiria, Portugal; 161 ALM – Serviços de Oftalmologia Médica e Cirúrgica, Lisbon, Portugal; 162 Hospital Garcia de Orta - ULS de Almada Seixal, EPE, Almada Portugal; 163 Instituto de Retina de Lisboa, Lisbon, Portugal; 164 UOC - Unidade de Oftalmologia de Coimbra S.A, Coimbra, Portugal; 165 Instituto de Microcirurgia Ocular (IMO), Lisbon, Portugal; 166 Unidade Local de Saúde Lisboa Ocidental - Hospital de Egas Moniz, Lisbon, Portugal; 167 MAGRABI Hospitals & Centers, Jeddah, Saudi Arabia; 168 King Khaled Eye Specialist Hospital, Riyadh, Saudi Arabia; 169 King Khalid University Hospital, Riyadh, Saudi Arabia; 170 Severance Hospital, Seoul, South Korea; 171 Hangil Eye Hospital, Incheon, South Korea; 172 Samsung Medical Center, Seoul, South Korea; 173 Kim’s Eye Hospital, Seoul, South Korea; 174 Pusan National University Hospital, Busan, South Korea; 175 Seoul National University Hospital, Seoul, South Korea; 176 YeungNam University Hospital, Daegu, South Korea; 177 Seoul National University Bundang Hospital, Seongnam, South Korea; 178 ChungNam National University Hospital, Daejeon, South Korea; 179 H Mérida, Mérida, Spain; 180 H León, León, Spain; 181 H San Pedro, Logroño, Spain; 182 H Terrassa, Terrassa, Spain; 183 H Donostia, San Sebastián, Spain; 184 HU Navarra, Pamplona, Spain; 185 H Son Llatzer, Palma de Mallorca, Spain; 186 HU Galdakao, Galdakao, Spain; 187 H Virgen Macarena, Seville, Spain; 188 CHU IMI Gran Canaria, Las Palmas, Spain; 189 HCU Lozano Blesa, Zaragoza, Spain; 190 H Civil Málaga, Málaga, Spain; 191 H Clinic Barcelona, Barcelona, Spain; 192 HU Bellvitge, L’Hospitalet de Llobregat, Llobregat, Spain; 193 H Cáceres, Cáceres, Spain; 194 H Son Espases, Palma de Mallorca, Spain; 195 H Naval Ferrol, Ferrol, Spain; 196 Hospital Miguel Servet, Zaragoza, Spain; 197 HU Burgos, Burgos, Spain; 198 H Esperança, Barcelona, Spain; 199 Ögonmottagning Falu Lasarett, Falun, Sweden; 200 Ögonläkarna i Eslöv AB, Eslöv, Sweden; 201 Sunderby Sjukhus, Ögonkliniken, Luleå, Sweden; 202 Skaane University Hospital Lund - Eye clinic A, Lund, Sweden; 203 Uppsala University Hospital - Eye Clinic, Uppsala, Sweden; 204 Swiss Visio Retina Research Center / Swiss Visio Montchoisi, Lausanne, Switzerland; 205 Hôpital ophtalmique Jules-Gonin, Lausanne, Switzerland; 206 Kantonsspital St. Gallen, Augenklinik (KSSG), St. Gallen, Switzerland; 207 Universitätsspital Zürich, Augenklinik USZ, Zürich, Switzerland; 208 Stadtspital Zürich Triemli, Augenklinik, Zürich, Switzerland; 209 Berner Augenklinik (Swiss Eye Institute), Bern, Switzerland; 210 Vista Augenklinik, Binningen, Switzerland; 211 Augenarzt Praxisgemeinschaft Gutblick AG, Bern, Switzerland; 212 LUKS Augenklinik, Lucerne, Switzerland; 213 Universitätsspital Bern, Klinik für Augenheilkunde, Bern, Switzerland; 214 Clinica di Oftalmologa (INSI), Ospedale Regionale di Lugano (EOC), Lugano, Switzerland; 215 Imperial College London - Al Ain Branch, Al Ain, United Arab Emirates; 216 Moorfields Eye Hospital Dubai, Dubai, United Arab Emirates; 217 Magrabi Eye Hospital – DHCC, Dubai, United Arab Emirates; 218 Cleveland Clinic Abu-Dhabi, Abu Dhabi, United Arab Emirates; 219 Kent and Canterbury Hospital, Canterbury, UK; 220 Bedford Hospital, Bedford, UK; 221 York Hospital, York, UK; 222 Worcestershire Royal Hospital, Worcester, UK; 223 Bristol Eye Hospital, Bristol, UK; 224 Gloucestershire Royal Hospital, Gloucester, UK; 225 University Hospital Southampton NHS Foundation Trust, Southampton, UK; 226 Royal Victoria Infirmary, Newcastle upon Tyne, UK; 227 Liverpool University Hospital, Liverpool, UK; 228 Hull University Teaching Hospitals NHS Trust, Hull, UK; 229 Frimley Health NHS Foundation Trust, Frimley, UK; 230 Sunderland Eye Infirmary, Sunderland, UK; 231 St. James’s University Hospital, Leeds, UK; 232 Wolverhampton Eye Infirmary, Wolverhampton, UK; 233 Western Eye Hospital, London, UK; 234 University Hospitals of Leicester, Leicester, UK

**Keywords:** Outcomes research, Retinal diseases, Drug therapy

## Introduction

The PHOTON Phase 2/3 trial demonstrated the efficacy and safety of intravitreal aflibercept 8 mg in the treatment of diabetic macular oedema (DME) [1]. SPECTRUM is a global study evaluating aflibercept 8 mg treatment of DME and neovascular age-related macular degeneration (nAMD) in routine clinical practice. This SPECTRUM analysis presents early clinical experience with aflibercept 8 mg among patients with treatment-naïve (TN) or previously treated (PT) DME.

## Methods

SPECTRUM (NCT06075147) is a 24-month, prospective observational study being conducted across 18 countries (February 2024–September 2027) in patients (TN or PT) with DME or nAMD. Each study site’s independent ethics committee/institutional review board approved the study protocol. Informed consent was collected from all participants.

Patients aged ≥18 years with DME and type 1 or type 2 diabetes mellitus, who had already been prescribed aflibercept 8 mg by their physician, were enrolled and treated per physician discretion and local clinical practice. Detailed enrolment criteria are reported at ClinicalTrials.gov [2]. This Week (W)8 analysis (visit closest to 56 [range: 43–70] days after baseline) was prespecified and included the first ~100 patients in each DME cohort to have a visit and visual acuity (VA) assessment at W8. All analyses are descriptive only.

## Results

**Fig. 1:**
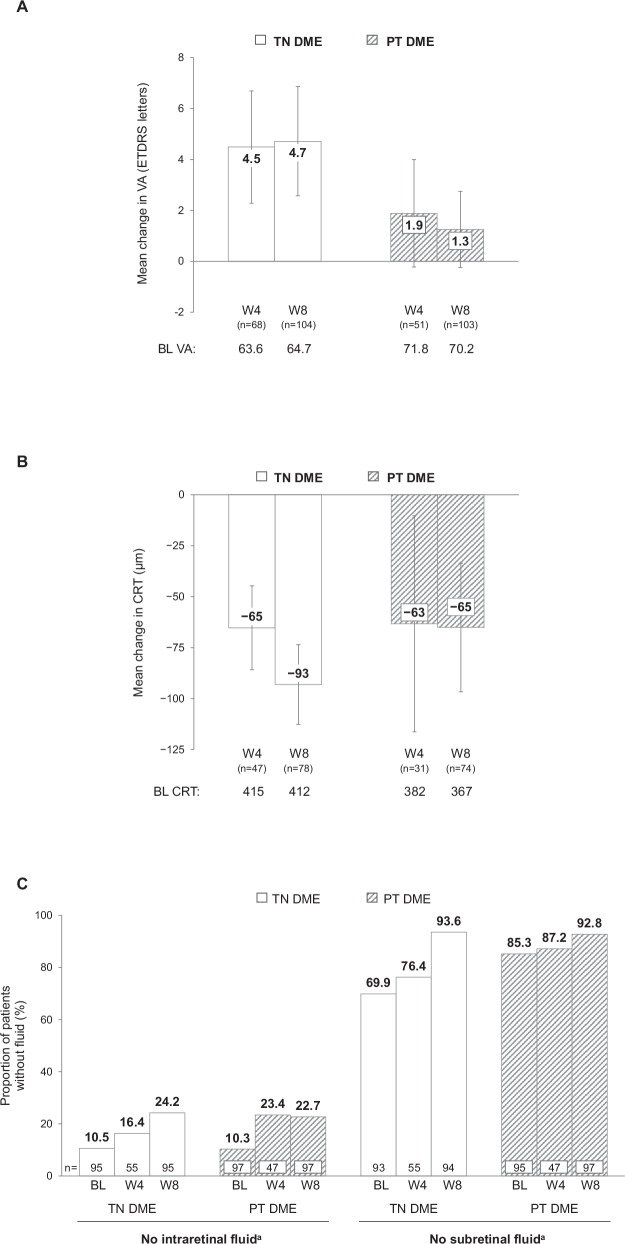
Functional and anatomical outcomes in patients with DME through Week 8 in SPECTRUM. **A** Mean change in VA from baseline through Week 8. **B** Mean change in CRT from baseline through Week 8. **C** Proportion of patients without intraretinal fluid or subretinal fluid through Week 8. Data are for the FAS (observed cases); error bars represent 95% CI. Mean VA/CRT change at Week 4 and Week 8 from baseline was calculated in patients with a VA/CRT assessment at Week 4 and Week 8, respectively. Week 4 = visits closest to 28 (14–42) days after the first injection (i.e., baseline), and Week 8 = visits closest to 56 (43–70) days after baseline. Note that outcomes described here would not reflect the effect of an injection received at Week 8, and some patients may not have received an injection at this timepoint. BL baseline, CI confidence interval, CRT central retinal thickness, DME diabetic macular oedema, ETDRS Early Treatment Diabetic Retinopathy Study, FAS full analysis set (all patients receiving ≥1 dose of study drug plus ≥1 post-baseline assessment), PT previously treated, TN treatment-naïve, VA visual acuity, W week. ^a^The presence of intraretinal fluid and subretinal fluid were determined by optical coherence tomography per physician discretion with the instrument available at each study site, and the proportions presented here were calculated based on the number of patients who had an assessment at each of the indicated time points.

Baseline characteristics are shown in Table 1. At W8 in the TN and PT DME cohorts, the mean (95% CI) change in VA was +4.7 (2.6, 6.9) and +1.3 (−0.2, 2.8) letters (Fig. 1A) from a baseline of 64.7 ± 16.3 and 70.2 ± 13.5 letters, and the mean (95% CI) change in central retinal thickness (CRT) was −93 (−113, −74) and −65 (−97, −34) µm (Fig. 1B) from a baseline of 412 ± 110 and 361 ± 141 µm. The proportions of patients without intraretinal fluid or subretinal fluid increased in both cohorts from baseline to W8 (Fig. 1C). Patients received a mean ± SD (median) of 2.7 ± 0.5 (3) and 2.5 ± 0.7 (3) injections through W8 in the TN and PT DME cohorts (first injection received at baseline). Note that the W8 injection data include any injections received at W8, whereas W8 outcomes reflect a response to injections administered before the W8 visit.

**Table 1:** Baseline demographics and disease characteristics of patients in the SPECTRUM Week 8 analysis of the treatment-naïve and previously treated DME cohorts.

	Treatment-naïve DME (*N* = 104)	Previously treated DME (*N* = 103)
Age, years	67.1 ± 10.1	65.5 ± 11.3
Female, *n* (%)	38 (36.5)	33 (32.0)
Race, *n* (%)^a^		
American Indian or Alaska Native	1 (1.0)	–
Asian	37 (35.6)	20 (19.4)
Black or African American	4 (3.9)	2 (1.9)
White	55 (52.9)	73 (70.9)
*Not reported*	7 (6.7)	8 (7.8)
Visual acuity, ETDRS letters^b^	64.7 ± 16.3	70.2 ± 13.5
Central retinal thickness, µm^c^	412 ± 110	361 ± 141
Median time (range) since DME diagnosis, months	0.5 (0.0, 93.6)	47.8 (3.3, 411.1)
Median duration (range) of prior DME treatment, days	–	865 (29, 5516)
Prior treatment for DME, *n* (%)		
Aflibercept 2 mg	–	62 (60.2)
Faricimab 6 mg	–	18 (17.5)
Ranibizumab 0.5 mg	–	11 (10.7)
Brolucizumab 6 mg	–	5 (4.9)
Other	–	5 (4.9)
Bevacizumab (variable dosage)	–	1 (1.0)
Steroids	–	1 (1.0)

FAS. Data are mean ± SD unless otherwise stated; percentages may not add up to 100 due to rounding.

*DME* diabetic macular oedema*,* *ETDRS* Early Treatment of Diabetic Retinopathy Study, *FAS* full analysis set (all patients receiving ≥1 dose of study drug plus ≥1 post-baseline assessment), *SD* standard deviation.

^a^Data on race were collected in Australia, Canada, Germany, Italy, Japan, Portugal, South Korea, Saudi Arabia, Spain, Switzerland, United Arab Emirates and the United Kingdom only; for France, Denmark, Finland, The Netherlands, Norway and Sweden, data on race were not collected according to local laws/regulations.

^b^Visual outcomes were assessed during routine clinical practice and reported in ETDRS letter scores; where ETDRS charts were unavailable, approximate Snellen scores were converted to ETDRS letter scores.

^c^Central retinal thickness was determined by optical coherence tomography per physician discretion with the instrument available at each study site.

In the TN and PT DME cohorts, ocular treatment-emergent adverse events (TEAEs) occurred in the study eye in 7/104 (6.7%) and 9/103 (8.7%) patients, and non-ocular TEAEs were observed in 6/104 (5.8%) and 5/103 (4.9%) patients. No new safety concerns were identified.

## Discussion

Results from this prespecified Week 8 analysis of SPECTRUM provide valuable insights into early real-world treatment response to aflibercept 8 mg in patients with DME. Patients received their first injection at baseline; by Week 8, both the TN and PT DME cohorts demonstrated improvements in VA and CRT, along with a reduction in retinal fluid. No new safety concerns were identified in this cohort.

As an observational study, the analyses from SPECTRUM are exploratory only. As this Week 8 analysis was based on only a subset of ~100 patients in each cohort, the outcomes in these global TN and PT DME cohorts will be more completely characterised once data become available for the full global cohorts.

Supplemental material is available at Eye’s website

## Data Availability

Availability of the data underlying this publication will be determined according to Bayer’s commitment to the European Federation of Pharmaceutical Industries and Associations/Pharmaceutical Research and Manufacturers of America (EFPIA/PhRMA) “Principles for responsible clinical trial data sharing.” This pertains to the scope, time point and process of data access. As such, Bayer commits to sharing upon request from qualified scientific and medical researchers’ patient-level clinical trial data, study-level clinical trial data and protocols from clinical trials in patients for medicines and indications approved in the United States of America (USA) and European Union (EU) as necessary for conducting legitimate research. This applies to data on new medicines and indications that have been approved by the EU and US regulatory agencies on or after 1 January 2014. Interested researchers can use www.clinicalstudydatarequest.com to request access to anonymised patient-level data and supporting documents from clinical studies to conduct further research that can help to advance medical science or improve patient care. Information on the Bayer criteria for listing studies and other relevant information is provided in the study sponsors' section of the portal. Data access will be granted for anonymised patient-level data, protocols and clinical study reports after approval by an independent scientific review panel. Bayer is not involved in the decisions made by the independent review panel. Bayer will take all necessary measures to ensure that patient privacy is safeguarded.
